# Are Founders More Socially Responsible? —An Empirical Research on Private Listed Companies in China

**DOI:** 10.3389/fpsyg.2021.707428

**Published:** 2021-08-16

**Authors:** Shi Xiaofei, Zhou Xiaoyu, Geng Lisha

**Affiliations:** School of Business Administration, Hebei University of Economics and Business, Shijiazhuang, China

**Keywords:** founder, founder management, founder's management level, corporate social responsibility, empirical research

## Abstract

Personal factors such as the founder's values and psychological characteristics will influence founder's vision, his perception and interpretation of the decision-making environment, and his strategic choice and decision-making. Based on the theory of entrepreneurial characteristics, combined with the founder's unique psychological characteristics, this paper takes Chinese private listed companies between 2010 and 2018 as a research sample to study the effect of the founder of private enterprises on corporate social responsibility. Furthermore, this study analyzes the impact of different management roles of the founder on corporate social responsibility. We demonstrate that the private enterprises have better social responsibility performance when there are founders; the founders have different management roles, and their corporate social responsibility performance has certain differences, and the higher the management level of the founders, the better the corporate social responsibility performance. This paper studies the issue of corporate social responsibility from the perspective of the characteristics of founders, which expands the current framework of corporate social responsibility research and provides an empirical basis for founders to effectively participate in corporate management in practice.

## Introduction

The issue of corporate social responsibility has long been a research topic which has received much attention from researchers. Corporate social responsibility affects the performance and development of the enterprise itself, also affects the economic growth and long-term stability of the whole society (Van Beurden and Gössling, 2008; Lenssen et al., 2011; Wang and Sarkis, 2017; Kong et al., 2020; Li et al., 2020). Due to the typical external characteristics of corporate social responsibility activities, in the short term, it may be more the increase of cost than the improvement of performance (Luo and Bhattacharya, 2006). Therefore, the current research on the influencing factors of corporate social responsibility focuses more on how to promote corporate social responsibility through the system, such as legal system (Gaint, 2010), media attention (Dyck et al., 2008; Saxton et al., 2019), moral culture (Ujan et al., 2020), and corporate internal governance mechanism (Li and Zhang, 2010), through various formal and informal systems to promote corporate social responsibility. However, even under the same system background, there are still great differences in the level of social responsibility and the focus of social responsibility among different enterprises, which indicates that the system is not the only way for enterprises to fulfill their social responsibility. According to the theory of entrepreneurial characteristics, entrepreneurs will have an important impact on the behavior and performance of enterprises. These characteristics dominate the thinking mode, decision-making habits, and action logic of entrepreneurs (Li, 2013), and have different impacts on the corporate strategy and operating performance (Wu and Wu, 2008), as well as the corporate social responsibility activities (Duan, 2011). Therefore, on the one hand, we should pay attention to external institutional factors, on the other hand, we should also take note of internal factors such as enterprise managers.

With the concern of the public on corporate social responsibility, Chinese enterprises are increasingly aware that corporate social responsibility behavior is conducive to the positive response of the public, which may have a positive effect on corporate performance and even form a competitive advantage (Biswas, 2019; Biswas and Tortajada, 2020). Therefore, enterprises will actively carry out corporate social responsibility strategic behavior to pursue greater profits (Flammer, 2015; Kaul and Luo, 2018). Its special personality and psychological characteristics enable the founder to have a broader vision and more accurate perception and deep interpretation of the decision-making environment than other managers, so as to promote the rapid development of the enterprise more effectively. The founder has made arduous efforts in the survival and development of the enterprise, and has a strong sense of responsibility and belonging to the enterprise. Therefore, when the founder participates in the management of the enterprise, compared with other senior management team members, the founder will use various ways to promote the development of the enterprise (Hu and Su, 2020). Mace (1985) and Pound (1995) have pointed out that founders and other senior managers have the power and responsibility to make decisions at the top of the organization. The founder has made outstanding contributions to the development of the company, has great influence and decision-making ability on the company, and has greater value. However, there is little literature on the relationship between founders, founder management, and corporate social responsibility.

**Figure 1:**
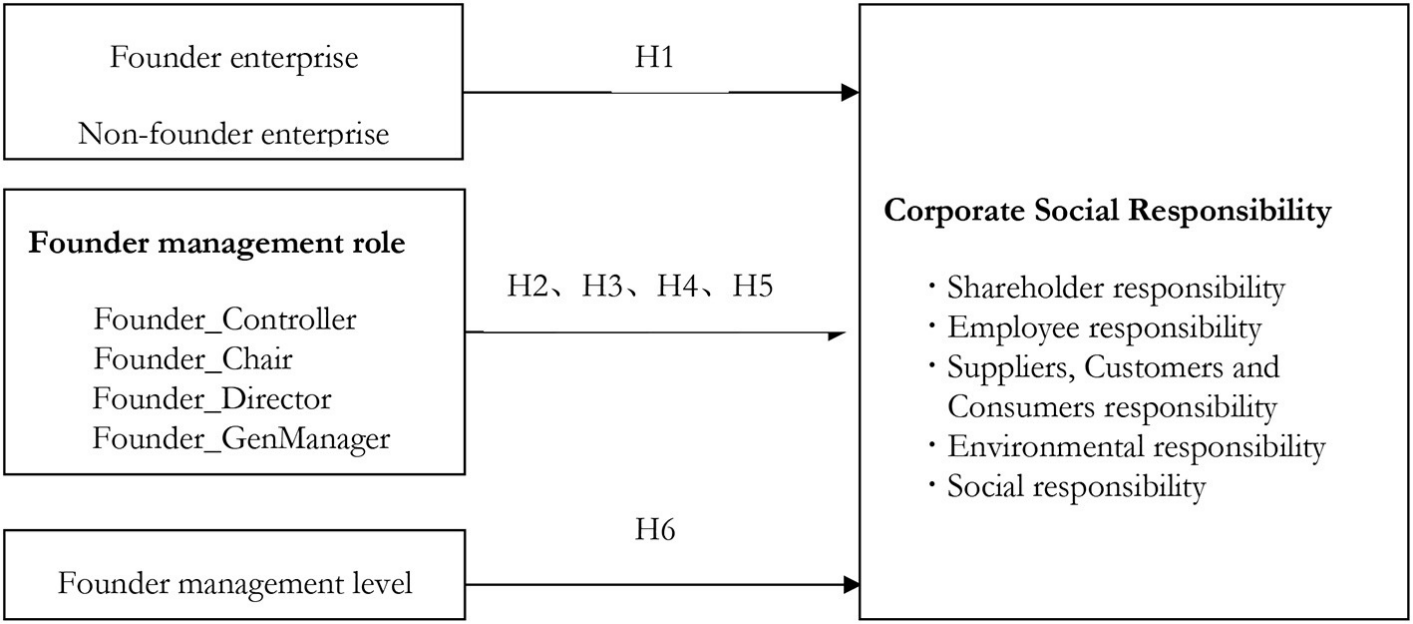
Conceptual Model of founder and corporate social responsibility.

Thus, this paper takes the founder of private enterprises as the starting point and analyzes the impact of the founders of private enterprises on corporate social responsibility according to the theory of entrepreneurial characteristics. Based on the previous analysis, we further analyzes whether the different management roles of founders affect corporate social responsibility (The research model of this paper is shown in Figure 1). These researches have the following important theoretical and practical significance: first, the research on the impact of founders on corporate social responsibility will help enrich the content of corporate social responsibility motivation research. At present, the research on the motivation of corporate social responsibility mainly focuses on the institutional motivation and economic motivation of corporate social responsibility, that is, it is generally believed that corporate social responsibility is for the system legally or to bring economic benefits to the enterprise. As a special manager in an enterprise, it will help to further understand the founders to clarify their motivation to fulfill their social responsibilities. Second, it will help to have a deeper understanding of the role of founder. As a special manager of an enterprise, the social responsibility consciousness of the founder of an enterprise is closely related to the development of the enterprise. From the perspective of the special management role of the founder of the enterprise, this paper studies the impact of the founder on corporate social responsibility and further explains the role of the founder in the corporate governance of private enterprises. Third, it provides the empirical basis for the founder to participate in the management of enterprises in practice. Based on the private enterprises with founders, this paper examines the influence of different management roles of founders on corporate social responsibility, which is of great significance to the management practice of private enterprises and provides inspiration for the formulation of relevant policies of private enterprises.

## Literature Review and Research Hypothesis

### Founders of Private Enterprises and Corporate Social Responsibility

The important reason for an enterprise to fulfill its social responsibility is to realize its profit and enterprise value (Flammer, 2015). The research found that corporate social responsibility can improve corporate reputation (Zhang et al., 2016), corporate financial performance (Wang et al., 2018), staff efficiency and emotional recognition (Tourigny et al., 2019). According to the theory of entrepreneurial characteristics, entrepreneurial behavior has a direct impact on the strategic decisions and operational activities of an enterprise. Therefore, entrepreneurial behavior is closely related to the fulfillment of the social responsibility of enterprises. The research of Wasserman (2003) and He (2008) pointed out that the special identity of the founder makes it easier to establish an emotional connection with the enterprise. Compared with other managers, the founder will strive to play his talents to promote the development of the enterprise. The close relationship between the founder and the enterprise enables the founder to put their various relationships and capital into the growth of the enterprise (Xu and Liu, 2012). Therefore, compared with other managers, the founder has a long-term and unique vision for the development of the enterprise, which makes them more inclined to pursue long-term interests rather than focusing on short-term actions or simply ensuring the stable profitability of the enterprise, thus providing a guarantee for improving the scientific decision-making and operating performance of the enterprise (Duchesneau and Gartner, 1990), and ensuring the growth and profitability of the enterprise. The founder's characteristics make the founder pay more efforts for the enterprise, carry out strategic corporate social responsibility behavior to pursue greater profits (Flammer, 2015; Kaul and Luo, 2018), and make the enterprise develop more long-term and healthy. These research results show that when there are founders in private enterprises, founders will pay more attention to corporate social responsibility to maintain their own property, long-term development, and reputation. Consequently, the following research assumptions are proposed:

**Hypothesis 1**. The founders of private enterprises have a positive effect on the overall performance of corporate social responsibility and have a positive effect on all dimensions of corporate social responsibility.

### Founder Management and Corporate Social Responsibility

As the initial framer of the organizational structure and development strategy, the special ability of the founder is very crucial to the sustainable growth of the enterprise (Nelson, 2003). Compared with the general managers, the founder has a stronger entrepreneurial spirit, can identify opportunities more effectively and be more willing to take risks (Johnson and Yi, 2013); in the decision-making process, the founder tends to respond more quickly to the market environment and have a long-term vision (Burgstaller and Wagner, 2015); moreover, the founder's hard work in the process of enterprise establishment and development will also endow him with a strong sense of mission and responsibility, and make him take less negative self-interest behaviors such as laziness and slowness (Wu and Hsu, 2018). Therefore, when the founder participates in the operation and decision-making of the enterprise, their special knowledge, experience, and organizational position will contribute to the long-term development of the enterprise (Johnson and Yi, 2013).

Founders are born with psychological ownership of the enterprises they founded (Wang et al., 2016). Therefore, founders often hold important management positions in the enterprises at the beginning of the establishment of the enterprises, such as the chairman, the CEO, or the chairman concurrently serving as the CEO. These positions are crucial to the enterprises, Pound (1995) pointed out that the CEO and other senior management has the power and responsibility for the senior decision-making of the organization within the organization. As the founder has made outstanding contributions to the development of the company, have great influence and decision-making ability on the company, and have greater value, therefore, when the founders are the senior management of the company, they have “natural advantages” (Fahlenbrach, 2009). Previous studies have shown that founders and other managers are not the same in terms of both internal characteristics and external incentives, which may have different impacts on the company's performance. When the founder participates in the company management, the connection between capital and non-capital gives the founder more enthusiasm and motivation to make better decisions and supervision. Therefore, the founder as a manager who actively participates in business management can significantly enhance the corporate value of the enterprise (Certo et al., 2001; Anderson and Reeb, 2003; Wu and Hsu, 2018). Xia et al. (2012) believes that in the long-term entrepreneurial process, the founder has accumulated a lot of management experience, authority and government relationship, so the impact of the founder management on the overall development of the enterprise will be more direct, and will certainly reflect the positive impact on the development of the enterprise. Li and Srinivasan studies have shown that when founders act as directors, there are more capital and non-capital connections that require founders to exercise supervision functions with better ability and motivation, and have a better governance environment than companies in which non-founders participate (Li and Srinivasan, 2011). Nelson (2003) pointed out that the founder as the general manager of the company has a positive effect on corporate governance and corporate development. This positive effect may come from the long-term incentive of the founders, in other word, the founders pay more attention to the long-term development of the enterprise rather than short-term performance, and may also come from the special assets of the operation and management of the enterprise owned by the founders themselves, such as reputation, experience, ability and relationship with the outside world. Therefore, when the founder participates in the enterprise management, for the development of the enterprise and the creation of a broader living space, the enterprise will take the initiative to undertake social responsibility, which is not only conducive to the establishment of the corporate image but also enables the sustainable development of the enterprise's society, economy and ecology (Hu, 2004). Consequently, we propose the following research assumptions:

**Hypothesis 2**. The founder of private enterprises as the actual controller has a positive effect on the overall performance of corporate social responsibility and has a positive effect on all dimensions of corporate social responsibility.
**Hypothesis 3**. The founder of private enterprises as the chairman of the board has a positive effect on the overall performance of corporate social responsibility and has a positive effect on all dimensions of corporate social responsibility.
**Hypothesis 4**. The founders of private enterprises as general managers have a positive effect on the overall performance of corporate social responsibility and have a positive effect on all dimensions of corporate social responsibility.
**Hypothesis 5**. The founder of private enterprises as a director has a positive effect on the overall performance of corporate social responsibility and has a positive effect on all dimensions of corporate social responsibility.

### Founder's Management Level and Corporate Social Responsibility

Based on the logical relationship of the impact of founder management on corporate social responsibility, it can be concluded that the level of founder management will also have an impact on corporate social responsibility. Since undertaking social responsibility can bring strategic benefits and reputation to the enterprise, the founder, as a special manager of the enterprise, can make its decision-making influence bigger by holding multiple management positions. The founder has accumulated rich professional knowledge and management experience in the development process of the company. The residual claim and residual control rights owned by the founder endow the founder with greater rights that guarantee a smooth advancement of their decision-making and increase their enterprise value. Donaldson believes that the ability of management can only be fully exerted if the power of management is not limited (Donaldson and Davis, 1991). If the general manager concurrently serves as the chairman of the board of directors, he will have stronger independence than other management, be more able to realize his own will, and have more power. Therefore, if the founder concurrently holds multiple management roles, the founder will have more stable power, which is conducive to the improvement of corporate value. Founder roles with different numbers of positions affect firms differently. Therefore, we select the number of important management roles held by founders to measure the management level of founders and propose the following research assumptions:

**Hypothesis 6**. The higher the management level of the founders of private enterprises, the positive effect on the overall performance of corporate social responsibility and the positive effect on all dimensions of corporate social responsibility.

## Materials and Methods

### Sample Selection

In 2009, in response to the requirements of “notice on strengthening the social responsibility undertaking of listed companies” issued by the Shanghai Stock Exchange, some listed companies in China successively disclosed independent social responsibility reports, which made it possible to obtain more systematic and comprehensive social responsibility information. Based on the social responsibility report of listed companies, Hexun.com began to evaluate the social responsibility performance of Listed Companies in 2010, and released data publicly, which provides the basis for this paper to measure the performance of corporate social responsibility. We were able to obtain data about CSR from 2010 and onwards. Hence, our sample consists of firms during 2010–2018. Due to the structural diversification of China's market economy and its unique institutional background, China's private economy has a broad and narrow sense. This paper draws on Wang Jinsong's narrow definition of the private economy (Wang et al., 2005). Thus, this study will selects enterprises other than state-owned and state-controlled enterprises and foreign-funded enterprises as the research sample. We also exclude financial and insurance listed companies because of the particularity of the business and accounting of financial and insurance companies in China. Finally, after excluding observations with incomplete data, a final sample with 10,119 firm-year observations is obtained. In the study of founder management, the enterprises without founders were excluded, and a total of 7,491 samples were obtained. The sample distribution is shown in Table 1.

**Table 1:** Sample distribution.

**Year**	**Total sample**	**Founder sample**
2010	758	514
2011	896	629
2012	956	676
2013	987	698
2014	1,050	756
2015	1,161	850
2016	1,303	984
2017	1,492	1,182
2018	1,516	1,202
Total	10,119	7,491

In this paper, the founder data is used to obtain the stock code of private listed companies according to the CSMAR database, and the company that is the founder is pointed out through the “information of the issuer” in the prospectus of the listed company, and through CSMAR database family business basic information database and Chinese listed company equity nature document database and Baidu.com search for auxiliary confirmation. Corporate social responsibility score related data from Hexun.com (http://stockdata.stock.hexun.com/) Social responsibility report of listed companies. Other data in this study are from the CSMAR database.

### Research Methodology

In order to test our hypotheses, we estimate the following models using OLS regression.

**Model 1**.

$$\begin{array}{l} CSR^{*}\;=\;\beta_{0}\;+\;\beta_{1}Founder+\gamma \;Control\;Variables_{jt}\\ \;+Industry\;dummies_{j}\;+\;Year\;dummies_{t}\;+\;\epsilon \end{array}$$

**Model 2**.

$$\begin{array}{l} CSR^{*}\;=\beta_{0}+\beta_{1}Fou\_Controller+\text{γ}Control\;Variables_{jt}\\ \;+Industry\;dummies_{j}\;+\;Year\;dummies_{t}\;+\;\epsilon \\ CSR^{*}\;=\;\beta_{0}+\beta_{1}Fou\_Chair\;+\text{ γ}Control\;Variables_{jt}\\ \;+Industry\;dummies_{j}\;+\;Year\;dummies_{t}\;+\;\epsilon \\ CSR^{*}\;=\beta_{0}\;+\;\beta_{1}Fou\_Director\;+\text{ γ}Control\;Variables_{jt}\\ \;+Industry\;dummies_{j}\;+\;Year\;dummies_{t}\;+\;\epsilon \\ CSR^{*}\;=\;\beta_{0}+\beta_{1}Fou\_GenManager\;+\text{ γ}Control\;Variables_{jt}\\ \;+Industry\;dummies_{j}\;+\;Year\;dummies_{t}+\epsilon \end{array}$$

**Model 3**.

$$\begin{array}{l} CSR^{*}\;=\;\beta_{0}\;+\;\beta_{1}Fou\_Manager+\text{γ}Control\;Variables_{jt}\\ \;+Industry\;dummies_{j}+Year\;dummies_{t}+\epsilon \end{array}$$

Among them, CSR^*^ is the score of corporate social responsibility, which are the total score of CSR_Total, shareholder responsibility score CSR_Shareholders, employee responsibility score CSR_Employees, supplier, customer and consumer responsibility score CSR_Suppliers, Customers, Consumers, environmental responsibility score CSR_Environmental and score CSR_Social.

### Dependent Variable

In these two models, the dependent variable is corporate social responsibility (CSR). In recent years, foreign kinds of literature mostly use the KLD index to measure, but there is no consistent method in China. In addition to using the well-known professional institution evaluation index KLD for reference, the domestic evaluation indicators of corporate social responsibility also use the scoring data of the third-party rating agencies for corporate social responsibility in China, such as Hexun.com social responsibility report professional evaluation system and Runling global organization for rating corporate social responsibility performance. Based on the research of Wang and Xu (2016) and Feng et al. (2016) this paper uses the social responsibility score of hexun.com professional evaluation system to measure the level of social responsibility of private enterprises. This score is based on the social responsibility report and financial report information of China's listed companies, which sets up 13 second-level indicators and 37 third level indicators, respectively from five aspects of shareholder responsibility, employee responsibility, supplier, customer and consumer responsibility, environmental responsibility, and social responsibility. The evaluation system systematically evaluates corporate social responsibility, which can reflect corporate social responsibility comprehensively and objectively. In recent years, it has been applied in more and more related researches.

### Explanatory Variables

The explanatory variables are founder, founder management and founder's management level. In this paper, referring to the relevant research of Xiaogang et al. (2011) and Xiaofei (2014), we define the founder as following the establishment and growth of the enterprise, relying on certain market opportunities and resources, relying on their organizational management ability, innovation consciousness, ability to identify and bear risks, and playing a role of actual control, interest coordinator, and risk-taking in the enterprise The final decision-maker and other important management roles. In this study, as long as the founder exists in the existing organizational structure of the company, we are identified as the founder company. In the process of founder confirmation, if the same enterprise has more than one founder, this article takes the founder with the highest position as the statistical object. It is defined as a binary variable and takes on two values: 1, indicating the existence of a founder in a private enterprise; otherwise 0.

We define founder management as the founder holding different management positions in the enterprise. After identifying the founder, we obtained the names of the actual controller, chairman, director, and general manager of the company from the CSMAR corporate governance database, and checked with the founder's name to determine whether the founder was the actual controller, chairman, director or general manager of the company. It is defined as a binary variable and takes on two values: 1, indicating the founder as actual controller of the company, otherwise 0; If the founder is the chairman of the company, the value is 1, otherwise 0; If the founder serves as general manager of the company, the value is 1, otherwise 0; If the serves as director of the company, the value is 1, otherwise 0.

We define founder's management level as the number of management positions held by the founder. It is defined as a categorical variable and takes on one of four values: 1, indicating the founder holds one management position; 2, indicating the founder holds two management positions; 3, indicating the founder holds three management positions; 4, indicating the founder holds four management positions; otherwise 0.

### Control Variables

In order to control the influence of other factors on the research conclusion, the following variables are selected as the control variables for the other main factors affecting the corporate social responsibility performance.

Firm Size. The research of Jia and Liu (2014) controlled the enterprise-scale and found that the enterprise-scale will affect the corporate social responsibility behavior.

Return on Assets (ROA). ROA equals operating profits divided by total assets. The higher the enterprise performance, the more likely the enterprise is to report its corporate social responsibility activities (Liao et al., 2018).

Asset-liability Ratio. Asset-liability Ratio equals total liabilities divided by total assets. which is used to control the impact of the capital structure of listed companies (Jia and Zhang, 2010).

Largest Shareholder Ratio. Based on Xia Lijun's literature, this paper selects the shareholding ratio of the first largest shareholder as the control variable, which represents the equity concentration (Flammer, 2018).

Total Assets Growth Rate. Total Assets Growth Rate refers to the growth of the enterprise's asset scale in the current period, reflecting the growth of the enterprise. Tian (2009) pointed out that corporate growth performance promotes corporate social responsibility.

Independent Director Ratio. Chen et al. (2015) believes that the board of directors with a higher proportion of independent directors improves the accounting information environment and improves the quality of financial reports, so independent directors can promote the implementation of corporate social responsibility and protect the interests of stakeholders (Fernández-Gago et al., 2016).

Board Size. Liao et al. (2018) believes that a large-scale board of directors can obtain different views from different stakeholders, and will invest more energy and resources to fulfill their roles in social activities and performance. Therefore, the larger size of the board of directors, the greater the possibility of enterprises voluntarily undertake social responsibility.

Separation of Ownership and Management. Separation of Ownership and Management refers to the difference between the control right and the ownership of the listed company owned by the actual controller. Yi et al. (2018) pointed out that the lower the separation of the two rights, the more conducive to the fulfillment of corporate social responsibility.

Firm Age. Referring to the research of Jia and Liu (2014), corporate age will affect corporate social responsibility behavior. This study controls the age of enterprises from the establishment of enterprises to 2018.

Year and Industry. In order to control the impact of uncertain factors at the macro-economic environment level and industry level on the corporate social responsibility performance, we use dummy variables to control the year and industry.

All variable definitions are shown in Table 2.

**Table 2:** Variable definitions.

	**Definition**
CSR_Total	Hexun's social responsibility for listed companies is mainly investigated from five aspects: shareholder responsibility, employee responsibility, supplier, customer and consumer rights and interests responsibility, environmental responsibility, and social responsibility
CSR_Shareholders	Hexun's responsibility to shareholders mainly measures profit, debt repayment, return, credit approval and innovation
CSR_Employees	Hexun's responsibility to employees mainly measures performance, safety and caring for employees
CSR_Suppliers, Customers, Consumers	Hexun's responsibility for the rights and interests of suppliers, customers, and consumers mainly measures product quality, after-sales service, and mutual trust
CSR_Environmental	Hexun's environmental responsibility mainly measures environmental governance
CSR_Social	Hexun's main measure of social responsibility is contribution value
Founder	The existence of a founder in a private enterprise, Fou equals 1, and 0 otherwise
Founder_Controller	If the founder is the actual controller of the company, Fou_con equals is 1, and 0 otherwise
Founder_Chair	If the founder is the chairman of the company, Fou_chi equals is 1, and 0 otherwise
Founder_Director	If the serves as director of the company, Fou_dir equals is 1, and 0 otherwise
Founder_GenManager	If the founder serves as general manager of the company, Fou_gen equals is 1, and 0 otherwise
Founder_Manager	The founder holds one management position, Fou_man equals is 1; The founder holds two management positions, Fou_man equals is 2; The founder holds three management positions, Fou_man equals is 3; The founder holds four management positions, Fou_man equals is 4; otherwise 0
Firm Size	The logarithm of a firm's total assets
Return On Assets	Operating profits divided by total assets
Asset-liability Ratio	Total liabilities divided by total assets
Largest Shareholder Ratio	The shareholding ratio of the largest shareholder
Total Assets Growth Rate	The growth of the enterprise's asset scale in the current period.
Board Size	The total number of directors in the board
Independent Director Ratio	The percentage of independent directors in the board
Separation of Ownership and Management	The difference between the control right and the ownership of the listed company owned by the actual controller
Firm Age	It is represented by the natural logarithm value of the number of years since a firm's inception
Industry	Dummy variable
Year	Dummy variable

## Results

Tables 3, 4 display the descriptive statistics of our sample. According to the descriptive statistics in Table 3, the average value of CSR_Total is 23.472, and the standard deviation is 14.988. According to the scoring method of Hexun, the total score of social responsibility should be 100, while the average score of social responsibility of private listed companies is 23.472, which indicates that the overall level of social responsibility of private listed companies in China is low, and there are differences in the level of social responsibility among different private enterprises. Regarding the variable of the founder, which accounts for 74% of the total sample. It shows that there are founders in most private enterprises. In Table 4, it can be seen that the actual controller of the founders accounts for 90% of the founder sample, and the founder chairman accounts for 74% of the founder sample. It shows that most of the founders participate in the management of the enterprise with the positions of actual controller and chairman. The founder's participation in enterprise management as the actual controller or chairman of the board has an impact on the enterprise's management and decision-making. The founder's characteristics make him make decisions conducive to the development of the enterprise when participating in enterprise management. Therefore, the founder's participation in enterprise management as the actual controller or chairman of the board is more conducive to the implementation of corporate social responsibility.

**Table 3:** Descriptive statistics (Founder).

	**Mean**	**Median**	**Std. dev**.	**Min**	**Max**
CSR_Total	23.472	21.7	14.988	−17.19	89.01
CSR_Shareholders	13.981	14.72	6.477	−13.12	27.92
CSR_Employees	2.301	1.35	2.712	−0.02	15
CSR_Suppliers, Customers, Consumers	1.328	0	4.229	0	20
CSR_Environmental	1.243	0	4.184	0	30
CSR_Social	4.619	4.11	4.452	−15	30
Founder	0.74	1	0.438	0	1
Independent Director Ratio	0.375	0.333	0.052	0.333	0.667
Largest Shareholder Ratio	32.886	30.73	14.609	2.38	95.95
Asset-liability Ratio	0.404	0.392	0.204	0.007	0.996
Total Assets Growth Rate	0.291	0.128	1.084	−0.957	47.927
Firm Size	21.803	21.728	1.185	10.897	26.739
Board Size	8.352	9	1.525	4	18
Return On Assets	0.044	0.042	0.26	−2.834	22.005
Firm Age	15.795	16	5.93	1	60
Separation of Ownership and Management	5.501	0.488	7.87	0	59.45

**Table 4:** Descriptive statistics(Founder management).

	**Mean**	**Median**	**Std. dev**.	**Min**	**Max**
CSR_Total	23.76	21.99	14.315	−15.23	89.01
CSR_Shareholders	14.802	15.52	6.155	−11.69	27.92
CSR_Employees	2.197	1.31	2.63	−0.02	15
CSR_Suppliers, Customers, Consumers	1.242	0	4.137	0	20
CSR_Environmental	1.186	0	4.167	0	30
CSR_Social	4.334	3.74	3.917	−15	30
Founder_Controller	0.901	1	0.299	0	1
Founder_Chair	0.747	1	0.435	0	1
Founder_Director	0.122	0	0.328	0	1
Founder_GenManager	0.324	0	0.468	0	1
Founder_Manager	2.094	2	0.87	0	4
Independent Director Ratio	0.375	0.333	0.053	0.333	0.667
Largest Shareholder Ratio	34.231	32.73	14.427	4.53	95.95
Asset-liability Ratio	0.376	0.362	0.191	0.008	0.989
Total Assets Growth Rate	0.295	0.144	0.681	−0.896	23.817
Firm Size	21.781	21.68	1.04	10.897	26.298
Board Size	8.342	9	1.484	4	18
Return On Assets	0.048	0.046	0.267	−2.834	22.005
Firm Age	14.444	14	5.753	1	43
Separation of Ownership and Management	4.676	0	7.447	0	59.45

In this study, Pearson test was used to analyze the correlation of founder, founder management degree, corporate social responsibility and other related variables. The detailed results are shown in Table 5. The research shows that there is the correlation among various variables, which preliminarily indicates that there is an internal relationship between variables, which can be further studied. In order to prevent the multicollinearity problem between variables, the VIF test is performed on variables. The multicollinearity test shows that the variance expansion factor VIF of all independent variables is less than the empirical critical value of 10, indicating that the variables are reasonable.

**Table 5:** Correlation matrix.

	**(1)**	**(2)**	**(3)**	**(4)**	**(5)**	**(6)**	**(7)**	**(8)**	**(9)**	**(10)**	**(11)**	**(12)**
1.CSR_Total	1											
2.CSR_Shareholders	0.620^***^	1										
3.CSR_Employees	0.752^***^	0.150^***^	1									
4.CSR_Suppliers, Customers, Consumers	0.799^***^	0.150^***^	0.813^***^	1								
5.CSR_Environmental	0.760^***^	0.125^***^	0.821^***^	0.878^***^	1							
6.CSR_Social	0.533^***^	0.279^***^	0.160^***^	0.203^***^	0.104^***^	1						
7.Founder	0.032^***^	0.214^***^	−0.064^***^	−0.034^***^	−0.023^**^	−0.108^***^	1					
8.Founder_Controller	0.068^***^	0.260^***^	−0.056^***^	−0.019^*^	−0.004	−0.092^***^	0.838^***^	1				
9.Founder_Chair	0.064^***^	0.257^***^	−0.065^***^	−0.029^***^	−0.010	−0.080^***^	0.658^***^	0.725^***^	1			
10.Founder_Director	0.010	0.032^***^	−0.008	−0.004	−0.013	0.008	0.187^***^	0.134^***^	−0.125^***^	1		
11.Founder_GenManager	0.010	0.140^***^	−0.050^***^	−0.040^***^	−0.037^***^	−0.067^***^	0.332^***^	0.367^***^	0.447^***^	−0.034^***^	1	
12.Founder_Manager	0.060^***^	0.270^***^	−0.070^***^	−0.035^***^	−0.022^**^	−0.093^***^	0.775^***^	0.867^***^	0.839^***^	0.231^***^	0.686^***^	1
13.Independent Director Ratio	−0.026^***^	−0.048^***^	0.002	−0.002	−0.018^*^	−0.001	−0.001	−0.008	0.008	−0.010	0.078^***^	0.026^**^
14.Largest Shareholder Ratio	0.143^***^	0.262^***^	−0.007	0.013	−0.009	0.101^***^	0.155^***^	0.188^***^	0.176^***^	0.039^***^	0.113^***^	0.199^***^
15.Asset-liability Ratio	−0.046^***^	−0.311^***^	0.123^***^	0.068^***^	0.078^***^	0.084^***^	−0.236^***^	−0.243^***^	−0.224^***^	−0.041^***^	−0.167^***^	−0.261^***^
16.Total Assets Growth Rate	0.036^***^	0.064^***^	0.025^**^	0	0.005	0.009	0.007	0.001	0.016	−0.001	0.026^***^	0.016
17.Firm Size	0.264^***^	0.170^***^	0.223^***^	0.165^***^	0.159^***^	0.200^***^	−0.030^***^	−0.029^***^	−0.056^***^	0.006	−0.090^***^	−0.066^***^
18.Board Size	0.112^***^	0.075^***^	0.082^***^	0.086^***^	0.078^***^	0.063^***^	−0.011	0.005	0.007	0.001	−0.072^***^	−0.021^**^
19.Return On Assets	0.114^***^	0.206^***^	0.016^*^	0.017^*^	0.012	0.047^***^	0.025^**^	0.023^**^	0.030^***^	−0.003	0.020^**^	0.028^***^
20.Firm Age	−0.056^***^	−0.131^***^	−0.006	−0.037^***^	−0.051^***^	0.088^***^	−0.385^***^	−0.394^***^	−0.351^***^	−0.039^***^	−0.197^***^	−0.385^***^
21.Separation of Ownership and Management	0.113^***^	0.044^***^	0.101^***^	0.097^***^	0.089^***^	0.078^***^	−0.177^***^	−0.164^***^	−0.165^***^	0.005	−0.157^***^	−0.190^***^
	**(13)**	**(14)**	**(15)**	**(16)**	**(17)**	**(18)**	**(19)**	**(20)**	**(21)**			
**Correlation matrix**															
13.Independent Director Ratio	1											
14.Largest Shareholder Ratio	0.029^***^	1										
15.Asset-liability Ratio	−0.009	−0.023^**^	1									
16.Total Assets Growth Rate	−0.012	0.008	−0.026^***^	1								
17.Firm Size	−0.045^***^	0.099^***^	0.389^***^	0.060^***^	1							
17.Board Size	−0.575^***^	−0.043^***^	0.079^***^	0	0.166^***^	1						
19.Return On Assets	−0.002	0.046^***^	−0.052^***^	0.022^**^	−0.030^***^	0.004	1					
20.Firm Age	0.022^**^	−0.150^***^	0.190^***^	−0.030^***^	0.120^***^	−0.017^*^	−0.027^***^	1				
21.Separation of Ownership and Management	−0.086^***^	0.222^***^	0.107^***^	−0.023^**^	0.154^***^	0.105^***^	0.003	0.054^***^	1			
	**1**	**2**	**3**	**4**	**5**	**6**	**7**					
**Phi Coefficients**																
1.Founder	1											
2.Founder_Controller	0.838^***^	1										
3.Founder_Chair	0.658^***^	0.421^***^	1									
4.Founder_Director	0.187^***^	−0.043^***^	−0.335^***^	1								
5.Founder_GenManager	0.172^***^	0.172^***^	0.322^***^	−0.104^***^	1							
6.Industry	0.355^***^	0.146^***^	0.102^***^	0.061^**^	0.105^***^	1						
7.Year	0.089^***^	0.138^***^	0.119^***^	0.057^***^	0.051^**^	1.005^***^	1					

^***^, ^**^, ^*^ represent the significance level of 1%, 5%, 10%, respectively.

In this paper, the sample is divided into six groups according to whether there is a founder, whether the founder is the actual controller, chairman, general manager and director of the enterprise, and the management level of the founder. The one-way ANOVA is used to test the level of corporate social responsibility among the groups. The statistical results are shown in Table 6.

**Table 6:** ANOVA analysis results.

	**CSR_Total**		**CSR_Shareholders**		**CSR_Employees**		**CSR_Suppliers, customers, consumers**		**CSR_Environmental**		**CSR_Social**	
	**Mean**	**P**	**Mean**	**P**	**Mean**	**P**	**Mean**	**P**	**Mean**	**P**	**Mean**	**P**
Non-Founder	22.65	0.001	11.64	0.000	2.60	0.000	1.24	0.001	1.19	0.019	4.33	0.000
Founder	23.76		14.80		2.20		1.57		1.41		5.43	
Total	23.47		13.98		2.30		1.33		1.24		4.62	
Non-Founder_Controller	19.79	0.000	11.45	0.000	2.23	0.696	1.27	0.055	0.77	0.004	4.33	0.754
Founder_Controller	24.20		15.17		2.19		0.96		1.23		4.38	
Total	23.76		14.80		2.20		1.24		1.19		4.33	
Non-Founder_Chair	22.05	0.000	12.80	0.000	2.36	0.002	1.32	0.356	1.20	0.492	4.30	0.156
Founder_Chair	24.34		15.48		2.14		1.22		1.13		4.44	
Total	23.76		14.80		2.20		1.24		1.19		4.33	
Non-Founder_Director	23.74	0.706	14.83	0.381	2.19	0.700	1.24	0.824	1.20	0.351	4.28	0.001
Founder_Director	23.93		14.63		2.23		1.27		1.07		4.73	
Total	23.76		14.80		2.20		1.24		1.19		4.33	
Non-Founder_GenManager	23.74	0.940	14.42	0.000	2.26	0.002	1.34	0.002	1.29	0.002	4.45	0.000
Founder_GenManager	23.77		15.60		2.06		1.03		0.97		4.09	
Total	23.76		14.80		2.20		1.24		1.19		4.33	
Non-Founder_Manager	17.69	0.000	10.63	0.000	1.95	0.000	0.66	0.000	0.52	0.000	3.93	0.000
One-Founder_Manager	22.99		12.86		2.59		1.53		1.51		3.60	
Two-Founder_Manager	23.59		15.30		2.27		1.45		1.38		4.45	
Three-Founder_Manager	24.19		15.51		1.98		1.32		1.43		4.16	
Four-Founder_Manager	24.90		15.65		2.25		0.93		0.87		4.64	
Total	23.76		14.80		2.20		1.24		1.19		4.33	

It can be seen from Table 6 that the average value of corporate social responsibility of private enterprises with founders is higher, and it passes the test at the 1% level, indicating that private enterprises with founders have more sense of social responsibility, which is consistent with our previous analysis. When the founder plays the role of enterprise management, the study finds that when the founder acts as the actual controller, chairman, general manager and director of the enterprise, the average value of corporate social responsibility is higher than that when the founder does not play the role of management, and it passes the inspection at the level of 1% and 10%, indicating that the private enterprise has better social responsibility performance when the founder plays the role of enterprise management, When the founder acts as the actual controller and chairman of the company, the sense of corporate social responsibility is stronger. From the perspective of Founder management, the higher the level of Founder management, the higher the average social responsibility, and passed the test at 1%, indicating that the higher the level of Founder management, the better the performance of corporate social responsibility.

First of all, based on whether there are founders in private enterprises, private enterprises are divided into founder enterprises and non-founder enterprises. In this part, this paper tests whether the founder of enterprise existence has a sense of social responsibility to verify Hypothesis 1.

Table 7 reports the regression results of corporate social responsibility of founders. It can be seen from Table 7 that the regression coefficient of the CSR_Total variable is 2.035, and it has passed the 1% significance level (β = 2.035, *p* < 0.01), indicating that the private enterprises with founders have better social responsibility performance, and Hypothesis 1 has been verified. The main reason is that the founders are more entrepreneurial than the other managers, more responsive to the market environment, and have a long-term vision. Therefore, the founders are more willing to encourage enterprises to engage in social responsibility activities than the other managers. Therefore, private enterprises with founders have a better realization of social responsibility. Column (2) to Column (6) of Table 7 examines the impact of founders on shareholder responsibility, employee responsibility, supplier, customer and consumer responsibility, environmental responsibility, and social responsibility. The results show that the regression coefficient of CSR_Shareholders, CSR_Suppliers, Customers, Consumers, and CSR_Environmental are 1.444, 0.253 and 0.196, and are significant at the level of 1%, 5%, and 10%, which indicates that the founders can better fulfill the shareholder responsibility, supplier, customer, and consumer responsibility and environmental responsibility. The relationship between founders' responsibility to employees and social responsibility is not significant, indicating that the founder pays insufficient attention to these two indicators. Through the test of grading indicators, the results show that founders prefer to perform external corporate social responsibility, which may be because the performance of external social responsibility will increase the goodwill of external stakeholders to the enterprise and have a positive impact on the company value. The results also reflect that the founders' understanding of corporate social responsibility may be more external.

**Table 7:** Regression results (founder and CSR).

	CSR^*^					
	**CSR_Total**	**CSR_Shareholders**	**CSR_Employees**	**CSR_Suppliers, customers, consumers**	**CSR_Environmental**	**CSR_Social**
Founder	2.035^***^	1.444^***^	−0.018	0.253^**^	0.196^*^	0.160
	(5.46)	(9.62)	(−0.26)	(2.27)	(1.77)	(1.48)
Independent Director Ratio	8.914^***^	−1.441	2.757^***^	3.976^***^	2.048^**^	1.574^*^
	(2.85)	(−1.14)	(4.70)	(4.27)	(2.20)	(1.73)
Largest Shareholder Ratio	0.069^***^	0.084^***^	−0.007^***^	−0.009^***^	−0.015^***^	0.016^***^
	(6.92)	(21.16)	(−3.67)	(−3.20)	(−5.19)	(5.43)
Asset-liability Ratio	−14.20^***^	−12.49^***^	−0.062	−0.343	0.002	−1.312^***^
	(−18.22)	(−39.76)	(−0.42)	(−1.47)	(0.01)	(−5.78)
Total Assets Growth Rate	0.032	0.202^***^	−0.018	−0.090^**^	−0.061	−0.001
	(0.26)	(4.00)	(−0.75)	(−2.42)	(−1.63)	(−0.02)
Firm Size	4.425^***^	1.713^***^	0.572^***^	0.764^***^	0.761^***^	0.614^***^
	(32.79)	(31.51)	(22.59)	(18.98)	(18.95)	(15.62)
Board Size	0.510^***^	0.168^***^	0.068^***^	0.123^***^	0.071^**^	0.080^**^
	(4.61)	(3.76)	(3.29)	(3.73)	(2.15)	(2.48)
Return On Assets	5.581^***^	4.357^***^	0.226^**^	0.172	0.115	0.710^***^
	(10.92)	(21.17)	(2.35)	(1.13)	(0.76)	(4.77)
Firm Age	0.082^***^	−0.019^*^	0.013^**^	0.030^***^	0.019^**^	0.039^***^
	(2.90)	(−1.66)	(2.48)	(3.53)	(2.24)	(4.75)
Separation of Ownership and Management	0.090^***^	0.004	0.022^***^	0.029^***^	0.025^***^	0.010^*^
	(4.90)	(0.52)	(6.41)	(5.32)	(4.53)	(1.91)
Industry	Control	Control	Control	Control	Control	Control
Year	Control	Control	Control	Control	Control	Control
Constant	−66.84^***^	−20.10^***^	−9.176^***^	−11.87^***^	−13.13^***^	−12.56^***^
	(−16.64)	(−12.42)	(−12.17)	(−9.91)	(−10.98)	(−10.74)
*N*	10119	10119	10119	10119	10119	10119
*R* ^2^	0.230	0.331	0.171	0.139	0.124	0.260

^***^, ^**^, ^*^ represent the significance level of 1%, 5%, 10%, respectively.

Secondly, for the development and control of the enterprise, founders often have absolute residual claim rights and residual control right over the enterprise. For the development and control of the enterprise, founders often have absolute residual claim rights and residual control right over the enterprise. They have a stronger desire for profit and the greatest degree of operational autonomy. Therefore, it is inevitable for the founder to play a core management role in the enterprise. In order to further explore the impact of different positions of founders on corporate social responsibility, Hypothesis 2, Hypothesis 3, Hypothesis 4, and Hypothesis 5 were tested. We divide the sample into: the founder serves as the actual controller, chairman, director or general manager of the listed company, that is, the value is 1, otherwise it is 0.

Table 8 reports the regression results of corporate social responsibility when the founder is the actual controller. According to Table 8, the regression coefficient of the CSR_Total is 2.564 and passed 1%Significance level (β = 2.564, *p* < 0.01), indicating that the private enterprises have better social responsibility performance when the founder is the actual controller, and Hypothesis 2 is verified. The reason lies in: firstly, when the founder is the actual controller of the enterprise, his interests tend to be consistent with the overall interests of the company. In order to enhance his interests and corporate value, the founder will be more active in fulfilling social responsibilities; In addition, the founder fulfills their social responsibilities, which is conducive to maintaining the stability of their control. If founders reduce their social responsibility, they will lose the support of stakeholders and related resources, which will affect the allocation of corporate control, and thus affecting the stability of the control of founders. Therefore, the founder will actively perform social responsibility to maintain its control. Column (2) to Column (6) of Table 8 examines the impact of indicators of social responsibility grading, and the results showed that the regression coefficient of CSR_ Shareholders is 2.513, which was significantly correlated at the level of 1%, while Founder_Controller has no significant effect on other social responsibility indicators. It indicates that the actual controller of the founder is more inclined to perform the shareholder's responsibility and pays less attention to other indicators of social responsibility, which may be the founder has inherent new ownership of the enterprise he founded. As the highest shareholder of the enterprise, the actual controller of the founder will pay more attention to the shareholder's responsibility and safeguard his interests when performing the social responsibility. In addition, for corporate shareholders, the performance of other social responsibilities is an additional part of the enterprise, which will cause losses to the interests of shareholders. Therefore, the actual controllers of founders prefer to perform their shareholder responsibilities.

**Table 8:** Regression results (founder actual controller and CSR).

	CSR^*^					
	**CSR_Total**	**CSR_Shareholders**	**CSR_Employees**	**CSR_Suppliers, customers, consumers**	**CSR_Environmental**	**CSR_Social**
Founder_Controller	2.564^***^	2.513^***^	−0.154	−0.033	0.056	0.181
	(4.89)	(11.84)	(−1.54)	(−0.20)	(0.35)	(1.33)
Independent Director Ratio	8.778^**^	0.787	2.029^***^	2.945^***^	1.416	1.601^*^
	(2.46)	(0.54)	(2.99)	(2.72)	(1.29)	(1.73)
Largest Shareholder Ratio	0.055^***^	0.070^***^	−0.004^*^	−0.008^**^	−0.011^***^	0.007^**^
	(4.89)	(15.31)	(−1.79)	(−2.24)	(−3.07)	(2.57)
Asset-liability Ratio	−16.05^***^	−13.65^***^	−0.468^**^	−0.326	−0.025	−1.583^***^
	(−16.58)	(−34.86)	(−2.54)	(−1.11)	(−0.08)	(−6.29)
Total Assets Growth Rate	0.405^*^	0.760^***^	−0.066	−0.185^***^	−0.198^***^	0.093
	(1.81)	(8.38)	(−1.55)	(−2.71)	(−2.86)	(1.60)
Firm Size	4.048^***^	1.460^***^	0.656^***^	0.762^***^	0.775^***^	0.394^***^
	(22.42)	(20.01)	(19.13)	(13.94)	(13.93)	(8.40)
Board Size	0.422^***^	0.231^***^	0.014	0.064	0.028	0.084^**^
	(3.23)	(4.38)	(0.58)	(1.62)	(0.69)	(2.49)
Return On Assets	4.709^***^	3.656^***^	0.221^**^	0.174	0.176	0.482^***^
	(8.43)	(16.19)	(2.09)	(1.03)	(1.02)	(3.32)
Firm Age	0.092^***^	0.003	0.013^**^	0.034^***^	0.024^***^	0.019^**^
	(3.07)	(0.21)	(2.25)	(3.69)	(2.59)	(2.45)
Separation of Ownership and Management	0.117^***^	0.013	0.020^***^	0.037^***^	0.032^***^	0.015^***^
	(5.42)	(1.48)	(4.88)	(5.73)	(4.77)	(2.67)
Industry	Control	Control	Control	Control	Control	Control
Year	Control	Control	Control	Control	Control	Control
Constant	−66.89^***^	−20.33^***^	−11.60^***^	−12.90^***^	−13.95^***^	−8.126^***^
	(−12.19)	(−9.16)	(−11.12)	(−7.75)	(−8.25)	(−5.70)
N	7491	7491	7491	7491	7491	7491
R^2^	0.213	0.304	0.159	0.135	0.119	0.291

^***^, ^**^, ^*^ represent the significance level of 1%, 5%, 10%, respectively.

Table 9 reports the founder chairman's return to corporate social responsibility. According to Table 9, the regression coefficient of the CSR_Total is 1.873, and passed the 1% significance level (β = 1.873, *p* < 0.01), indicating that the founder would better perform social responsibilities when he served as the chairman, and Hypothesis 3 was verified. The reason lies in that, as the owner of the enterprise, the personal interests of the founder and the chairman are highly consistent with the interests of the enterprise. In order to make the enterprise established by himself develop for a long time, the founder and the chairman of the board attach importance to the long-term development strategy of the enterprise, so the founder and the board of directors better perform their social responsibilities. Column (2) to Column (6) of Table 9 examines the impact of social responsibility grading indicators test shows that the regression coefficient of CSR_Shareholders is 2.513 and was significantly correlated at the 1% level, whereas the regression coefficient of CSR_Employees is −0.173 and was significantly correlated at the 5% level, Founder_Chair and other indicators are not significant, indicating that the founder and chairman of the board are more inclined to perform the responsibilities of shareholders and less inclined to perform the responsibilities of employees for corporate social responsibility. This may be because the founder and chairman of the board will make business decisions based on the principle of maximizing their interests, and such business results will better meet the interests of shareholders. The reason for the negative correlation between the founder and the chairman of the board and employee responsibility, as well as other social responsibilities, is that: firstly, the performance of employee responsibility and other social responsibilities is a cost expense for the enterprise and will reduce the interests of shareholders; In addition, when the founder and the chairman of the board have a high degree of confidence, they will underestimate the importance of employees and other stakeholders for the development of the enterprise and do not pay attention to the performance of employee responsibilities and other social responsibilities.

**Table 9:** Regression results (Founder and Chairman with CSR).

	CSR^*^					
	**CSR_Total**	**CSR_Shareholders**	**CSR_Employees**	**CSR_Suppliers, customers, consumers**	**CSR_Environmental**	**CSR_Social**
Founder_Chair	1.873^***^	2.054^***^	−0.173^**^	−0.152	−0.008	0.151
	(5.20)	(14.14)	(−2.52)	(−1.39)	(−0.07)	(1.62)
Independent Director Ratio	8.189^**^	0.147	2.082^***^	2.989^***^	1.417	1.553^*^
	(2.29)	(0.10)	(3.07)	(2.76)	(1.29)	(1.67)
Largest Shareholder Ratio	0.054^***^	0.068^***^	−0.004^*^	−0.007^**^	−0.010^***^	0.007^**^
	(4.81)	(14.99)	(−1.65)	(−2.10)	(−3.02)	(2.52)
Asset-liability Ratio	−16.08^***^	−13.63^***^	−0.480^***^	−0.356	−0.037	−1.580^***^
	(−16.63)	(−34.99)	(−2.61)	(−1.21)	(−0.12)	(−6.29)
Total Assets Growth Rate	0.355	0.711^***^	−0.063	−0.184^***^	−0.199^***^	0.090
	(1.58)	(7.88)	(−1.49)	(−2.71)	(−2.88)	(1.54)
Firm Size	4.106^***^	1.515^***^	0.653^***^	0.763^***^	0.777^***^	0.398^***^
	(22.84)	(20.92)	(19.13)	(14.01)	(14.02)	(8.52)
Board Size	0.395^***^	0.201^***^	0.017	0.067^*^	0.028	0.082^**^
	(3.03)	(3.82)	(0.69)	(1.69)	(0.70)	(2.42)
Return On Assets	4.682^***^	3.628^***^	0.223^**^	0.175	0.176	0.480^***^
	(8.39)	(16.13)	(2.11)	(1.04)	(1.02)	(3.31)
Firm Age	0.094^***^	0.006	0.012^**^	0.033^***^	0.024^**^	0.020^**^
	(3.13)	(0.49)	(2.16)	(3.60)	(2.56)	(2.49)
Separation of Ownership and Management	0.121^***^	0.018^**^	0.019^***^	0.036^***^	0.031^***^	0.015^***^
	(5.59)	(2.08)	(4.72)	(5.58)	(4.72)	(2.74)
Industry	Control	Control	Control	Control	Control	Control
Year	Control	Control	Control	Control	Control	Control
Constant	−66.07^***^	−19.54^***^	−11.64^***^	−12.90^***^	−13.93^***^	−8.069^***^
	(−12.05)	(−8.84)	(−11.18)	(−7.76)	(−8.24)	(−5.66)
N	7491	7491	7491	7491	7491	7491
R^2^	0.214	0.310	0.159	0.136	0.119	0.291

^***^, ^**^, ^*^ represent the significance level of 1%, 5%, 10%, respectively.

Table 10 reports the regression results of the founder's general manager on corporate social responsibility. As can be seen from Table 10, the regression coefficient of the CSR_Total variable is 0.575, and passed the 10% significance level (β = 0.575, *p* < 0.1), indicating that the founder would better perform social responsibilities when he served as the general manager, and Hypothesis 4 was verified. The reason is that when the founder is the general manager of the enterprise, his behavior affects the strategic decision-making and implementation of the company to a large extent. As the manager of the enterprise, the interests of the founder general manager tend to converge with the interests of the shareholders of the company, so that the founder general manager makes strategic decisions conducive to the long-term development of the enterprise. Therefore, the founder general manager will better perform the corporate social responsibility. Column (2) to Column (6) of Table 10 examines the impact of social responsibility classification shows that the regression coefficient for CSR_Shareholders is 1.026 and is significantly correlated at the 1% level, whereas the regression coefficients of CSR_Suppliers, Customers, Consumers and CSR_Environmental are −0.171 and −0.170, and are significantly correlated at the 10% level, Founder_GenManager and other grading indicators are not significant, indicating that the founder general manager is more inclined to perform shareholder responsibilities and pays less attention to other social responsibilities, especially the rights and interests of suppliers, customers and consumers, and environmental responsibilities. The reason may be: first of all, as the doer of the principle of “self-interest,” the founder general manager will give priority to the maximization of his value in the implementation of strategic decisions, so the attention to other social responsibilities is not high. In addition, the founder general manager may pursue short-term interests in the operation of the enterprise, therefore, his awareness of the rights and responsibilities of suppliers, customers and consumers, and environmental responsibility is poor.

**Table 10:** Regression results (founder general manager and CSR).

	CSR^*^					
	**CSR_Total**	**CSR_Shareholders**	**CSR_Employees**	**CSR_Suppliers, customers, consumers**	**CSR_Environmental**	**CSR_Social**
Founder_GenManager	0.575^*^	1.026^***^	−0.079	−0.171^*^	−0.170^*^	−0.030
	(1.73)	(7.59)	(−1.26)	(−1.70)	(−1.66)	(−0.35)
Independent Director Ratio	8.405^**^	0.159	2.077^***^	3.043^***^	1.512	1.614^*^
	(2.35)	(0.11)	(3.06)	(2.81)	(1.37)	(1.74)
Largest Shareholder Ratio	0.059^***^	0.072^***^	−0.004^*^	−0.007^**^	−0.001^***^	0.008^***^
	(5.25)	(15.73)	(−1.82)	(−2.09)	(−2.86)	(2.76)
Asset-liability Ratio	−16.43^***^	−13.95^***^	−0.452^**^	−0.347	−0.063	−1.621^***^
	(−17.01)	(−35.57)	(−2.46)	(−1.19)	(−0.21)	(−6.47)
Total Assets Growth Rate	0.339	0.685^***^	−0.061	−0.180^***^	−0.195^***^	0.090
	(1.51)	(7.51)	(−1.44)	(−2.65)	(−2.83)	(1.55)
Firm Size	4.139^***^	1.559^***^	0.650^***^	0.758^***^	0.774^***^	0.399^***^
	(22.98)	(21.31)	(19.01)	(13.91)	(13.96)	(8.54)
Board Size	0.432^***^	0.241^***^	0.014	0.064	0.028	0.085^**^
	(3.31)	(4.55)	(0.56)	(1.62)	(0.69)	(2.51)
Return On Assets	4.690^***^	3.632^***^	0.223^**^	0.176	0.178	0.481^***^
	(8.39)	(16.00)	(2.10)	(1.04)	(1.03)	(3.32)
Firm Age	0.084^***^	−0.004	0.013^**^	0.033^***^	0.023^**^	0.018^**^
	(2.80)	(−0.32)	(2.31)	(3.66)	(2.52)	(2.36)
Separation of Ownership and Management	0.113^***^	0.012	0.020^***^	0.036^***^	0.030^***^	0.014^**^
	(5.21)	(1.41)	(4.84)	(5.53)	(4.53)	(2.52)
Industry	Control	Control	Control	Control	Control	Control
Year	Control	Control	Control	Control	Control	Control
Constant	−66.10^***^	−19.63^***^	−11.64^***^	−12.87^***^	−13.89^***^	−8.056^***^
	(−12.03)	(−8.80)	(−11.16)	(−7.75)	(−8.22)	(−5.65)
N	7491	7491	7491	7491	7491	7491
R^2^	0.211	0.297	0.159	0.136	0.119	0.291

^***^, ^**^, ^*^ represent the significance level of 1%, 5%, 10%, respectively.

Table 11 shows the return of founder directors to corporate social responsibility. According to Table 11, the overall performance of Founder_Director and social responsibility is not significant, and the social responsibility of each dimension is not significant, which indicates that when the founder serves as the corporate director, the corporate does not have better social responsibility performance, and Hypothesis 5 has not been verified. The reason may be that when the founder is a corporate director, the impact of the founder on the corporate strategic decision-making and implementation is not significant, therefore, the founder as a corporate director has no significant corporate social responsibility.

**Table 11:** Regression results (founder director and CSR).

	CSR^*^					
	**CSR_Total**	**CSR_Shareholders**	**CSR_Employees**	**CSR_Suppliers, customers, consumers**	**CSR_Environmental**	**CSR_Social**
Founder_Director	0.134	−0.308	0.122	0.083	−0.010	0.246^**^
	(0.29)	(−1.64)	(1.40)	(0.59)	(−0.07)	(2.07)
Independent Director Ratio	8.741^**^	0.721	2.041^***^	2.951^***^	1.415	1.614^*^
	(2.44)	(0.49)	(3.01)	(2.73)	(1.29)	(1.74)
Largest Shareholder Ratio	0.061^***^	0.075^***^	−0.004^**^	−0.008^**^	−0.011^***^	0.008^***^
	(5.46)	(16.58)	(−1.97)	(−2.28)	(−3.05)	(2.73)
Asset–liability Ratio	−16.53^***^	−14.12^***^	−0.438^**^	−0.319	−0.035	−1.615^***^
	(−17.13)	(−35.92)	(−2.40)	(−1.09)	(−0.12)	(−6.46)
Total Assets Growth Rate	0.351	0.708^***^	−0.063	−0.184^***^	−0.199^***^	0.089
	(1.56)	(7.74)	(−1.48)	(−2.71)	(−2.88)	(1.53)
Firm Size	4.128^***^	1.538^***^	0.652^***^	0.761^***^	0.777^***^	0.400^***^
	(22.93)	(20.97)	(19.08)	(13.98)	(14.02)	(8.57)
Board Size	0.432^***^	0.241^***^	0.014	0.064	0.028	0.085^**^
	(3.30)	(4.52)	(0.56)	(1.62)	(0.70)	(2.52)
Return On Assets	4.697^***^	3.640^***^	0.223^**^	0.175	0.176	0.483^***^
	(8.40)	(15.97)	(2.10)	(1.03)	(1.02)	(3.33)
Firm Age	0.083^***^	−0.006	0.013^**^	0.034^***^	0.024^**^	0.018^**^
	(2.75)	(−0.53)	(2.33)	(3.70)	(2.58)	(2.35)
Separation of Ownership and Management	0.108^***^	0.005	0.020^***^	0.037^***^	0.031^***^	0.014^**^
	(5.03)	(0.62)	(4.95)	(5.73)	(4.75)	(2.47)
Industry	Control	Control	Control	Control	Control	Control
Year	Control	Control	Control	Control	Control	Control
Constant	−65.99^***^	−19.44^***^	−11.65^***^	−12.91^***^	−13.93^***^	−8.065^***^
	(−12.01)	(−8.68)	(−11.18)	(−7.77)	(−8.24)	(−5.66)
N	7491	7491	7491	7491	7491	7491
R^2^	0.211	0.291	0.159	0.135	0.119	0.292

^***^, ^**^, ^*^ represent the significance level of 1%, 5%, 10%, respectively.

Through the above empirical research results, it is found that the founders' different management roles have a positive impact on corporate social responsibility, but different management positions have different impacts on corporate social responsibility. The regression results show that the founder as the actual controller, chairman, and general manager can better perform corporate social responsibility than as a director. The role of the founder as the actual controller, chairman, and general manager has a greater impact on the strategic decision-making and implementation of the strategy of the enterprise, thus, the enterprise has a better performance of social responsibility. While the role of the founder as a director has little influence on the enterprise, therefore, the impact of the founder director on corporate social responsibility is not significant. From the regression results, the founders as the actual controller, the chairman, and the general manager are more inclined to perform the responsibilities of shareholders, which is because the founder themselves, like corporate shareholders, adhere to the principle of “egoism,” and are more inclined to pursue long-term wealth creation for shareholders. This kind of interest orientation urges the founders to have more enthusiasm and motivation to make better decisions and supervision for the enterprise. However, for the founder, fulfilling other social responsibilities is a costly expense for the enterprise in the short term, which will damage their interests and the interests of corporate shareholders. Therefore, the founder does not pay much attention to other social responsibilities.

At the end of this paper, we examine the impact of the founder's management level on corporate social responsibility. Based on the positive impact of different management positions held by founders on corporate social responsibility, this section examines the impact of founder management on corporate society. Some researchers have pointed out that the more positive the attitude of senior managers toward corporate social responsibility, the better the economic performance of their enterprises (Sturdivant and Ginter, 1977). In the long-term entrepreneurial process, the founders have accumulated a lot of operation and management experience, authority and the relationship with government departments. Therefore, when the founder manages the enterprise, it is conducive to the development of the enterprise. The founder who holds multiple management roles at the same time will have more stable power, which is conducive to the improvement of corporate value.

Table 12 shows the regression results of the impact of the founder's management level on corporate social responsibility. In this table, the regression coefficient of the CSR_Total variable is 0.989, and passed the 1% significance level (β = 0.989, *p* < 0.01), indicating that the higher the founders' management level, the better the enterprise will perform its social responsibility and Hypothesis 6 is verified. Because when the founder holds multiple management positions, his decision-making influence becomes greater. The higher the power of the founder, the more he can ensure the smooth progress of decision-making and improve the enterprise value. When the power of the founder is not limited, the founder's ability can be fully exerted. Therefore, if the founder holds multiple management roles at the same time, the founder will have a more stable power and be more conducive to corporate social responsibility. Column (2) to Column (6) of Table 12 examines the impact on social responsibility classification shows that the regression coefficients of CSR_Shareholders and CSR_Suppliers, Customers, Consumers are 1.095 and 0.091, and are significantly correlated at the 1% and 10% levels, while the regression coefficient of CSR_Employees is −0.068, which is significantly correlated at the level of 10%, Founder_Manager and other grading indicators are not significant, indicating that the higher the management level of founders, the more likely they are to perform their shareholder responsibilities and social responsibilities and pay insufficient attention to other social responsibilities, especially to employee responsibilities. The reasons may be as follows: firstly, the higher the management level of the founders, the greater their rights, and the stronger the founders' sense of belonging to the enterprise. The fulfillment of shareholders' and social responsibilities not only satisfies their interests but also brings a good reputation image to the enterprise, which is more conducive to the long-term development of the enterprise; secondly, the founder will make decisions according to their interests. The higher the management level of the founders, the greater their rights, and the greater the ability of the founders to obtain private benefits for themselves. In order to obtain more benefits for themselves, they pay less attention to other social responsibilities; finally, when the management level of the founder is higher, the founder may have more confidence in his own business decisions. At this time, when the founder makes business decisions, he will not consider too much social responsibility to ease the relationship between the company and stakeholders, to stabilize the development of the company. Therefore, the founder will not pay too much attention to employee responsibility and other social responsibilities.

**Table 12:** Regression results (founder's management level and CSR).

	CSR^*^					
	**CSR_Total**	**CSR_Shareholders**	**CSR_Employees**	**CSR_Suppliers, customers, consumers**	**CSR_Environmental**	**CSR_Social**
Founder_Manager	0.989^***^	1.095^***^	−0.068^*^	−0.082	−0.048	0.091^*^
	(5.41)	(14.89)	(−1.95)	(−1.48)	(−0.85)	(1.92)
Independent Director Ratio	7.970^**^	−0.102	2.084^***^	3.008^***^	1.452	1.527^*^
	(2.23)	(−0.07)	(3.07)	(2.78)	(1.32)	(1.65)
Largest Shareholder Ratio	0.051^***^	0.065^***^	−0.004	−0.007^**^	−0.010^***^	0.007^**^
	(4.55)	(14.26)	(−1.64)	(−2.02)	(−2.88)	(2.40)
Asset-liability Ratio	−15.94^***^	−13.47^***^	−0.479^***^	−0.368	−0.064	−1.563^***^
	(−16.45)	(−34.55)	(−2.60)	(−1.25)	(−0.21)	(−6.21)
Total Assets Growth Rate	0.351	0.707^***^	−0.063	−0.184^***^	−0.199^***^	0.090
	(1.57)	(7.84)	(−1.48)	(−2.71)	(−2.88)	(1.54)
Firm Size	4.107^***^	1.515^***^	0.653^***^	0.763^***^	0.778^***^	0.398^***^
	(22.85)	(20.95)	(19.11)	(14.01)	(14.04)	(8.52)
Board Size	0.410^***^	0.217^***^	0.015	0.066^*^	0.029	0.083^**^
	(3.14)	(4.13)	(0.62)	(1.66)	(0.72)	(2.45)
Return On Assets	4.693^***^	3.640^***^	0.222^**^	0.174	0.176	0.481^***^
	(8.41)	(16.20)	(2.10)	(1.03)	(1.02)	(3.31)
Firm Age	0.094^***^	0.006	0.013^**^	0.033^***^	0.023^**^	0.020^**^
	(3.13)	(0.50)	(2.21)	(3.60)	(2.51)	(2.50)
Separation of Ownership and Management	0.123^***^	0.021^**^	0.019^***^	0.036^***^	0.031^***^	0.016^***^
	(5.70)	(2.42)	(4.74)	(5.53)	(4.61)	(2.80)
Industry	Control	Control	Control	Control	Control	Control
Year	Control	Control	Control	Control	Control	Control
Constant	−66.58^***^	−20.10^***^	−11.61^***^	−12.86^***^	−13.90^***^	−8.116^***^
	(−12.14)	(−9.11)	(−11.14)	(−7.74)	(−8.22)	(−5.70)
N	7491	7491	7491	7491	7491	7491
R^2^	0.214	0.312	0.159	0.136	0.119	0.292

^***^, ^**^, ^*^ represent the significance level of 1%, 5%, 10%, respectively.

## Conclusion

Based on the sample of private listed companies in China, this paper uses the data of sample firms 2010–2018 to analyze the impact of founders of private enterprises on corporate social responsibility and further discusses the impact of different management roles of founders on social responsibility. The results show that: firstly, compared with private enterprises without founders, private enterprises with founders have more sense of social responsibility. Moreover, the existence of founders can promote enterprises to better fulfill the responsibilities of shareholders, suppliers, customers and consumers, and environmental responsibilities, but pay less attention to employee responsibilities and social responsibilities, which also reflects that founders' understanding of corporate social responsibility may be more externality. Therefore, the founders of private enterprises can better perform their social responsibilities. Secondly, the impact of founders' different management roles on corporate social responsibility is positive, but there are certain differences in the performance of corporate social responsibility. The founders as the actual controller, chairman, and general manager can better perform the corporate social responsibility than the directors, because the founders as the actual controller, chairman, and general manager have a greater influence on the enterprise, so their social responsibility performance is better. In addition, when the founder serves as the actual controller, chairman, and general manager, he pays more attention to shareholders' responsibility and protects his interests. Because the performance of other social responsibilities is a costly expense for the enterprise in the short term, which will damage its interests and the interests of corporate shareholders, the founder does not pay much attention to other social responsibilities. Finally, the impact of founder's management level on corporate social responsibility found that the higher the level of founder management, the stronger the sense of corporate social responsibility. The founder has more power, which can promote the improvement of enterprise value, and the greater the management power, the greater the decision-making power, and is more conducive to the development of the enterprise.

This study analyzes the impact of private enterprise founders on corporate social responsibility from the perspective of founders, enriches the research content of corporate social responsibility, and further deepens the understanding of the special management role of founders. At the same time, this research conclusion has enlightenment significance for founders to manage enterprises in practice. Therefore, the following management suggestions are proposed:

First, the vigorous implementation of social responsibility activities by the founders of private enterprises is conducive to maintaining the personal reputation of the founders, obtaining more benefits, and long-term sustainable development of the enterprises. Therefore, the founder of an enterprise should first perform his due responsibilities, and then try to assume more responsibilities for all stakeholders. Second, under the condition of limited resources, the founders can make targeted investment in more external corporate social responsibility, but also cannot ignore the internal responsibility when making decisions on corporate development strategies. Although the external corporate social responsibility has more public relations effect and weakens the negative impact of the internal and external social responsibility inconsistency on the corporate value; however, from the perspective of long-term foundation, employees are the real assets and wealth of the enterprise. The enterprise can motivate employees and achieve the goal of common development of employees and the organization by obtaining their internal recognition and appreciation. Third, we should give full play to the role of the founder in the enterprise, allow the founder to participate in the enterprise management, give more rights to the founder, and make it play a greater role in the enterprise operation and decision-making, to achieve long-term healthy development of the enterprise.

## Data Availability Statement

The original contributions presented in the study are included in the article/supplementary material, further inquiries can be directed to the corresponding author.

## Author Contributions

SX designed the paper, collected the data, summarized the literature review, developed the hypotheses, conducted the empirical analysis, and finalized the manuscript. ZX contributed to the hypothesis development and performed the empirical analysis. GL provided valuable suggestions and comments regarding the manuscript. All authors contributed to the article and approved the submitted version.

## Conflict of Interest

The authors declare that the research was conducted in the absence of any commercial or financial relationships that could be construed as a potential conflict of interest.

## Publisher's Note

All claims expressed in this article are solely those of the authors and do not necessarily represent those of their affiliated organizations, or those of the publisher, the editors and the reviewers. Any product that may be evaluated in this article, or claim that may be made by its manufacturer, is not guaranteed or endorsed by the publisher.
